# Radioactive iodine therapy: multiple faces of the same polyhedron

**DOI:** 10.20945/2359-3997000000461

**Published:** 2022-05-12

**Authors:** Rosália do Prado Padovani, Sumedha V. Chablani, Robert Michael Tuttle

**Affiliations:** 1 Faculdade de Ciências Médicas da Santa Casa de Misericórdia de São Paulo São Paulo SP Brazil Faculdade de Ciências Médicas da Santa Casa de Misericórdia de São Paulo, São Paulo, SP, Brasil; 2 Memorial Sloan Kettering Cancer Center New York NY United States Memorial Sloan Kettering Cancer Center, New York, NY, United States

## Abstract

The incidence of differentiated thyroid carcinoma (DTC) has increased in recent decades with early stage, low risk papillary thyroid cancer (PTC) being detected and diagnosed. As a result, the psychological, financial, and clinical ramifications of overdiagnosis and excessively aggressive therapy are being increasingly recognized with many authorities calling for a re-evaluation of the traditional “one size fits all” management approaches. To address these critical issues, most thyroid cancer guidelines endorse a more risk adapted management strategy where the intensity of therapy and follow up is matched to the anticipated risk of recurrence and death from DTC for each patient. This “less is more” strategy provides for a minimalistic management approach for properly selected patients with low-risk DTC. This has re-kindled the long-standing debate regarding the routine use of radioactive iodine therapy (RIT) in DTC. Although recent guidelines have moved toward a more selective use of RIT, particular in patients with low-intermediate risk DTC, the proper selection of patients, the expected benefit, and the potential risks continue to be a source of ongoing controversy and debate. In this manuscript, we will review the wide range of clinical, imaging, medical team, and patient factors that must be considered when evaluating individual patients for RIT. Through a review of the current literature evaluating the potential benefits and risks of RIT, we will present a risk adapted approach to proper patient selection for RIT which emphasizes peri-operative risk stratification as the primary tool that clinicians should use to guide initial RIT management recommendations.

## INTRODUCTION

For more than 50 years, the management of differentiated thyroid carcinoma (DTC) has included total thyroidectomy, radioactive iodine therapy (RIT), and levothyroxine suppressive therapy which is often referred to as a “one size fits all” management approach) (1).

Given the increased incidence of DTC in recent decades (2) mainly due to the detection of low-risk tumors, treatment goals and outcomes have been revised, with a focus on avoiding unnecessary therapy for patients (3). The benefits of routine use of RIT have come into question mainly for low- and intermediate-risk patients (4). Due to the lack of high-quality evidence comparing low-intensity with more aggressive treatments, mainly in low- and intermediate-risk patients, there are differing management approaches between centers. There are clinicians who advocate for low-intensity treatment options and those who strongly favor high-intensity treatment options (5,6).

**Figure 1:**
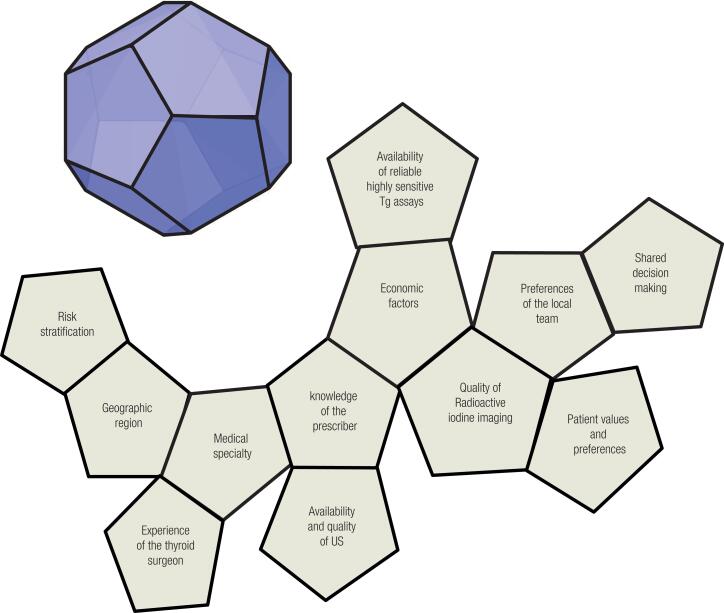
A multi-faced polyhedron showing the many factors beyond traditional risk stratification which have a major impact on whether or not RIT is recommended for individual patients.

Recent guidelines have recommended a more conservative and individualized treatment for tumors without suspicious or aggressive characteristics (4,7). However, the cost related to RIT in countries such as Brazil has been increasing, suggesting that a more selective approach to patient selection for RIT is not being widely adopted (8). It is also important to recognize that many factors beyond traditional risk stratification have a major impact on whether or not RIT is recommended for individual patients. These include factors such as a) geographic region, b) medical specialty, c) knowledge of the prescriber, d) economic factors, e) availability and quality of ultrasonography, f) quality of radioactive iodine imaging, g) experience of the thyroid surgeon, h) availability of reliable highly sensitive thyroglobulin assays, i) patient values and preferences, and j) preferences of the local disease management team, k) sharing decision making (4,9–12). So rather than thinking about RIT decision making as a simple triangle that integrates thyroid cancer risk with the risks and benefits of RIT, we envision this as a complex, shared decision making process that more resembles a multi-faced polyhedron (Figure 1) that requires the patient and the disease management team to integrate many different factors to arrive at the best management option for any individual patient being treated within their specific geographic, family, socioeconomic, and medical context.

Recognizing that the initial administered activity of RAI could be used for a variety of goals and purposes, a consensus group of United States and European thyroid and nuclear medicine associations recommended that the initial goals of RAI therapy be characterized as either remnant ablation (to destroy residual thyroid tissue remaining after surgical thyroidectomy), adjuvant treatment (to destroy microscopic residual disease that may or may not remain after initial thyroid surgery), or treatment of known disease (to treat RAI avid disease identified by biochemical, structural or functional imaging) using standardized definitions (12). This would facilitate ongoing discussions and allow potential risks and benefits of RAI to be evaluated within these three very different clinical contexts.

In this manuscript, we will review the wide range of factors that form the multiple faces of the polyhedron and examine how they can be integrated into an individualized, risk adapted approach to the management of patients with differentiated thyroid cancer. Ideally, this approach will match the intensity of therapy and follow-up to the anticipated risks associated with the cancer so that the minimum therapy necessary to achieve low recurrence rates and excellent overall survival are selected for individual patients.

## How to select a patient for RIT

### Peri-operative risk stratification

The corner stone of a risk adapted management approach is a careful and thoughtful evaluation of risk which begins at diagnosis and is continually modified over time as new data is accumulated (13,14). Initial staging can provide important information regarding the expected clinical outcomes. An understanding of the risk of disease-specific mortality and recurrence following initial surgical intervention is critical in determining whether additional therapies, such as RIT, are likely to have a substantial beneficial impact.

The Tumor-Node-Metastasis (TNM) staging system, developed by the American Joint Commission on Cancer (AJCC) and the Union for International Cancer Control (UICC), is the most used classification to assess the risk of disease-specific mortality (15). According to its latest edition (8^th^ edition AJCC), most patients with DTC (> 90%) are classified as stage I or II with the risk of death being < 2%.

To better estimate the risk of disease recurrence, the 2015 American Thyroid Association (ATA) guidelines (4) recommend the division of patients into three categories of recurrence risk (low, intermediate, and high) (Table 1) while recognizing that the risk of recurrence is probably better thought of as a continuum of risk rather than 3 discrete categories, which have been validated in several retrospective studies (13). The European Consensus Conference (ECC) suggests a similar classification system (very low, low, and high) (7). In fact, most guidelines base their recommendations for RIT on the ATA's and ECC's recommendations (16).

**Tabla 1:** Initial staging definition and the estimate of risk of persistent structural or recurrent disease in each category as per the ATA 2015 guideline

Initial staging	Definition	Risk estimate of structural disease recurrence
Low	Intrathyroidal DTC Clinical N0 or ≤5 pathologic N1 micro-metastases (<0.2 cm in largest dimension) No local or distant metastases All macroscopic tumor has been resected No tumor invasion of loco-regional tissues or structures No aggressive cyto-type (e.g., tall cell, hobnail variant, columnar cell carcinoma) If RAI was given, there are no RAI-avid metastatic foci outside the thyroid bed on the first post treatment WBS No vascular invasion Intra-thyroidal encapsulated follicular variant of papillary thyroid cancer Intra-thyroidal well differentiated follicular thyroid cancer with capsular invasion and no or minimal (<4 foci) vascular invasion	≤5%
Intermediate	Minor extrathyroidal extension **Presence of vascular invasion** **Tumor with aggressive histology (e.g., tall cell, insular, columnar cell carcinoma, Hurthle cell carcinoma, follicular thyroid cancer)** RAI-avid metastatic foci in the neck on the first post-treatment WBS Clinical N1 or >5 pathologic N1 with all involved LN <3 cm in largest dimension **Intrathyroidal papillary thyroid carcinoma, <4 cm, BRAFV600E mutated (if known)**	>5%-20%
High	Gross extrathyroidal extension – macroscopic Incomplete tumor resection Distant metastasis Postoperative serum thyroglobulin suggestive of distant metastasis Pathologic N1 with any metastatic LN ≥ 3 cm in largest dimension FTC with extensive vascular invasion (>4 foci of vascular invasion)	>20%

It is particularly important to emphasize that initial risk stratification should be based on all the clinical, imaging, and biochemical testing information available in the peri-operative period which extends from the time of diagnosis until 4 months after initial surgery (11,14). This would include not only the pre-operative evaluations, but information from the surgeon's intra-operative findings, the histopathology report, the post-operative thyroglobulin, and any imaging either structural or functional obtained in the first 4 months after surgery (11,14). Integration of all this information allows the clinicians to provide reliable risk estimates with regard to risk of dying from thyroid cancer (AJCC stage), risk of having disease recurrence (ATA risk) and initial response to therapy assessments, intra-operative findings, post-op thyroglobulin, and post-op imaging if clinically indicated. From a practical standpoint, we usually recommend RAI therapy be given after this evaluation considering also patient preference and the practice pattern of the local disease management team.

### Postoperative evaluation

#### Potential utility of cervical ultrasound after surgery

In experienced hands, it is the most valuable test to detect thyroid remnants, metastases from locoregional disease, and thyroid bed metastases in the pre- or post-operative period (17). Neck US is a non-invasive test, relatively easy to perform, cost-effective, and can guide diagnostic and therapeutic procedures with a low rate of complications. However, the main limitation of this exam is that it is highly dependent on scanning equipment and protocols. In addition, it is operator-dependent and therefore must be performed by an experienced radiologist (12)

From a practical perspective, routine use of ultrasound in the immediate post-operative period (<4 months after initial surgery) is not necessary in low to intermediate risk patients that had appropriate pre-operative imaging and evaluations, complete resection of thyroid gland and thyroid cancer by an experienced thyroid surgeon, a normal post-operative physical examination and non-stimulated serum thyroglobulin < 5 ng/mL. However, Leenhardt and cols. have consistently demonstrated the utility of neck ultrasonography to detect thyroid remnants, metastases from locoregional disease, and thyroid bed metastases in the first several months after surgery (17). Thus, neck ultrasonography can be a valuable post-operative staging tool if there are clinical, biochemical, or physical examination findings suggesting inadequate surgical resection of the thyroid gland or thyroid cancer foci in the neck.

A post-operative mass in the thyroid bed is considered suspicious if it is hypoechoic and/or has a cystic component, calcifications, irregular shape or borders, or increased vascularization (4). US characteristics that suggest metastasis of thyroid cancer included the presence of calcification, cystic change, loss of echogenic fatty hilum, hyper-echogenicity, round shape, and abnormal vascularity (18). Of these, some studies have shown that calcification and cystic changes have 100% specificity and positive predictive value, and that they are not observed in normal or reactive LNs (19,20).

Consideration for additional surgery is warranted if post-operative imaging identifies persistent disease that is amenable to surgical resection without exposing the patient to undue risk. A multidisciplinary management approach integrating size of the metastatic foci, location of the disease, expected RAI avidity, and patient preference is needed to determine the best approach to a persistent disease. In general, a surgical approach is favored over RAI or external beam irradiation therapy if the surgery can be done without excessive risks or morbidity.

#### Is diagnostic RAI scanning needed to facilitate the initial staging?

Although pre-ablation whole body scan (WBS) was historically performed to complete patient evaluation prior to RIT, in recent years, its clinical utility has been a controversial issue. In consideration of questions concerning its benefits and the possibility of it causing stunning effects, physicians prefer the use of an empirical, fixed dose for RAI therapy based on the histopathologic results of the tumor (7,21,22).

Diagnostic WBS can be performed with different iodine isotopes (^123^I, ^124^I, ^131^I) and acquisition modalities (e.g., planar SPECT, SPECT/CT, PET/CT) with varying sensitivities and specificities. ^123^I is predominantly a gamma emitter with better physical qualities for images than standard gamma cameras. For pre-ablation WBS preformed with this radioisotope, higher administered activities can be used compared to ^131^I with less concern for stunning. A disadvantage of ^123^I is that it is more expensive and is not as readily available as ^131^I. Also, a diagnostic ^123^I scans when compared to ^131^I post treatment scans have been shown to underestimate the disease burden especially in children and in other patients who have had prior RAI therapy and/or distant metastasis (11).

^124^I (a positron-emitting tomography tracer), in combination with computed tomography (PET/CT) has made possible the detection of thyroid cancer lesions with high sensitivity and resolution besides providing data for 3D radiobiology and dosimetry. Santhanam and cols., in a systematic review and meta-analysis demonstrated that ^124^I PET/CT is a sensitive tool to diagnose radioiodine avid DTC lesions, and that it also detects many lesions that are not visualized on the post-treatment ^131^I scan (23). From a technical perspective, the ^124^I PET/CT image can offer clinical advantages, since it offers superior imaging characteristics with improved spatial resolution and image sensitivity (11).

A diagnostic pre-ablation WBS may assist in the identification of iodine-avid regional and/or distant metastases and thus in the determination of the amount of iodine activity to be administered. Van Nostrand and cols. reported that 53% of patients have findings in pre-ablation WBS that may alter the management of their disease. Chen and cols. also reported that the pre-ablation I-123 WBS can provide additional critical information in 25% of patients, which alters the therapeutic strategy (24).

SPECT/CT has been used in the management of patients with DTC. SPECT/CT has some advantages over conventional whole body planar scintigraphy, as it provides better image quality and localization of abnormal foci of RAI accumulation, positively impacting staging, risk stratification, and overall patient care (11) although it is associated with increased radiation exposure, additional imaging time, and increased costs over routine planar imaging. However, recent work has shown that there is a benefit of SPECT/CT when combined with pre-ablation WBS in improving image interpretation and post-surgical staging of DTC (25,26). SPECT/CT can help in the detection of LN and distant metastases and in differentiating between benign and malignant physiological activity. Avram and cols. demonstrated that SPECT/CT associated with pre ablation WBS was able to detect residual or unsuspected regional metastases in 35% of cases and distant metastases in 8% of cases in 320 consecutive patients, which changed the initial staging based only on clinical information and histopathological findings in 4% of younger patients and 25% of older patients (26). Rager and cols. concluded that the combination of WBS and SPECT/CT changed the initial diagnosis in 12 out of 212 patients (5.7%) and increased the detection rate of extra-axial lesions, particularly in the femoral neck (the area associated with the highest risk of pathologic fractures) (27). Pre-ablation SPECT/CT combined with stimulated serum thyroglobulin (sTg) levels appears to be even more predictive. Avram and cols. showed that together, WBS, SPECT/CT and sTg performed after surgery and before ^131^I treatment altered the initial risk stratification in 15% of patients, which led to changes in the clinical management strategy in 29.4% of patients (28). Together, these modalities can assist in the selection of which patients will (or will not) benefit from RIT and on RAI dosing (29,30).

Recently, a joint statement from the American Thyroid Association, the European Association of Nuclear Medicine, the European Thyroid Association, and the Society of Nuclear Medicine and Molecular Imaging carefully review the role of diagnostic RAI imaging in intermediate risk patients (11). Jointly, they supported a selective use approach diagnostic RAI scanning in ATA intermediate risk patients rather than routine use or denial for all patients in this category. They noted that peri-operative risk stratification could be improved with diagnostic RAI imaging and therefore could provide important management information for properly selected patients. Thus, the final decision regarding diagnostic RAI imaging will rely on clinicians and patients’ judgement as to whether this information would significantly impact management recommendations when integrated into all the other information being considered as part of the polyhedron decision matrix.

If metastases are detected, the prescribed ^131^I activity can be increased or a dosimetry calculation can be performed to improve the therapeutic efficacy of RIT. As the therapeutic effect depends on the radiation absorbed dose, the elimination of regional or distant metastases requires administration of higher ^131^I activity. For LN metastases, for example, Maxon and cols. (31) demonstrated that regional or distant metastases can be successfully eliminated just when at least 8,000 rad (80 Gy) are delivered to tumors.

On the other hand, RIT for structurally identifiable disease that is not RAI-avid by diagnostic scanning is very unlikely to have a significant tumoricidal effect and is therefore not generally recommended. Patients may be candidates for other types of procedures such as ethanol injection, radiofrequency, or laser ablation if they are high-risk surgical patients or in case of refusing additional surgery (4).

### Evaluation of post-operative Tg and TgAb

Postoperative Tg obtained at least 6 weeks after thyroid surgery is a critical factor in the composite peri-operative risk assessment that can guide decision-making regarding RIT. However, the precise Tg value, either basal or stimulated, that can be used to distinguish minimal residual normal tissue from persistent thyroid cancer has not been firmly established although we expect a non-stimulated thyroglobulin value obtained at least 6 weeks after surgery to be less than 5 ng/mL and often less than 2 ng/mL if a total thyroidectomy is done by an experienced thyroid surgeon (4,16). Following thyroid lobectomy, non-stimulated Tg values are expected to be < 30 ng/mL but usually are less than 10 ng/mL (32).

TgAb are present in up to 30% of patients with DTC and do not correlate with the tumor burden, but rather, with the tumor's immunological activity. TgAb interference can result in falsely low results in Tg immunoassays and falsely high or falsely low results in radioimmuno assays (33,34). In recent years, liquid chromatography with tandem mass spectrometry has emerged as a promising novel method to overcome interferences in the measurement of Tg but unfortunately, this method is not yet used routinely.

Serum TgAb has an average disappearance time of 3 years after thyroid ablation. In disease-free patients, a drop in TgAb of more than 50% is usually observed in the first year after initial treatment, which allows TgAb to be used in the follow-up of patients with DTC who have undergone total thyroidectomy and posterior RIT (34–37).

There is no data currently available to properly inform the management of TgAb-positive follow-up after lobectomy or thyroidectomy alone. Thus, a plausible recommendation is that patients with TgAb that are intermediate to high risk of recurrence can be considered for RAI to ablation to facilitate initial staging and follow-up. Alternatively, in low to intermediate risk subgroups, the trend in TgAb levels over time, without RAI ablation, can be used to guide dynamic risk stratification and ongoing management.

### What is the surgeon's experience and what type of surgery was performed?

The quality of the initial surgery, tumor histology, and disease extent are all factors that influence the risk of tumor recurrence (38). In the literature, most studies about the quality of the initial surgery are related to surgical complications, such as recurrent laryngeal nerve injury (39,40). However, complications do not necessarily reflect the adequacy of tumor resection and/or dissection of metastatic LNs (38). Thus, there is a lack of indicators related to surgical quality that are easily measured and specific for DTC.

Schneider and cols. suggested that the percentage of cervical uptake on postoperative RAI diagnostic scans and the LN ratio (the proportion of metastatic nodes to the total number of nodes dissected) can be used as potential quality indicators in surgery for DTC (41). A lower remnant percent uptake of RAI was significantly associated with surgeons with a large surgical volume (*P* = 0.002) and a lower complication rate (38).

Additionally, the number of surgeries performed each year are strong predictors of improved oncological outcomes and lower complication rates. A recent study from the European Society of Endocrine Surgery demonstrated significantly lower recurrence rates, a lesser need for re-operation and a lower rate of complications when the initial surgery was performed by high volume surgeons (>50 thyroidectomies per surgeon per year) when compared to low volume surgeons (< 25 thyroidectomies per surgeon per year) (42,43).

In conclusion in thyroid surgery a volume and outcome relationship exists with respect to the prevalence of complications. Besides volume, cumulative experience is expected to improve outcomes. In accordance with global data, a case load of < 25 thyroidectomies per surgeon per year appears to identify a low-volume surgeon, while > 50 thyroidectomies per surgeon per year identify a high-volume surgeon. A center with a case load of > 100 thyroidectomies per year is considered high-volume. Thyroid cancer and autoimmune thyroid disease predict an increased risk of surgical morbidity and should be operated by high-volume surgeons. Oncological results of thyroid cancer surgery are significantly better when performed by high-volume surgeons.

### What are the patient's preferences and values?

It is important to note that an increasing proportion of patients with thyroid cancer are showing a desire to understand and share in the decision-making with their physicians. Studies have suggested that medical information is one of the most important factors that influence patients’ decisions (44,45). Furthermore, patients value the shared decision-making process which enhances trust in their clinician, the treating institution and makes them feel that they were active participants in the decision-making process (46,47).

Therefore, it is the responsibility of the physician to explain the indications, benefits, and potential risks of RIT. Furthermore, the patients’ wishes, objections, hopes, and fears must be considered in the final treatment decision.

### The rationale of RIT

#### Low-risk DTC

Low-risk DTC patients are, by definition, patients who present a low risk of recurrent disease ≤ 5% (Table 1) according to the ATA 2015 guideline, and an even lower risk of cancer-related death (48). Although several staging systems can be used to assess mortality risk, the AJCC/TNM system from the UICC is the most widely used system in clinical practice. According to the criteria established by the 8th edition, patients classified as stage I have a 10-year survival of 99% and those classified as stage II have a 10-year survival of > 95% (15). The ATA 2015 guideline also recognized non-invasive follicular thyroid neoplasm with papillary-like nuclear features (NIFTP) as low-risk tumors. These tumors, previously classified as non-invasive encapsulated follicular variant of papillary thyroid cancer, have a low risk of recurrence (<1% in 15 years) (49). Patients with low-risk DTC normally live full, productive lives with no evidence of disease after initial treatment. As such, initial treatment should be selected in order to minimize any treatment related morbidities (16).

To date, there is little evidence to suggest that RAI can improve disease-free survival (DFS), disease specific survival or overall survival (OS) in low-risk patients (16), and there is conflicting evidence relating to the impact of RAI on the risk of recurrence (9,12,16,50–53). Two systematic reviews have not shown a significant impact of adjuvant ^131^I on disease-related mortality and have shown some conflicting results regarding the risk of recurrence (52,54). More recent data, using the risk classification criteria proposed by the ATA 2015 guideline, suggest the lack of significant impact of RIT on risk of recurrence in patients in this group (16). The initial results of ESTIMABL2 also demonstrated the non-inferiority of a conservative follow-up strategy compared to systematic adjuvant post-operative administration of RAI (1.1GBq following rh TSH) in low-risk DTC patients (55).

The ESTIMABL2 [NCT01837745] is a French multicentric randomized phase III trial in patients with low-risk DTC treated with total thyroidectomy with or without prophylactic neck LN dissection (pT1am N0 or Nx with a sum of the diameters of tumor lesions ≥ 10 mm, pT1b N0 or Nx). Two to 5 months after surgery, in the absence of suspicious lateral neck LNs on US, patients were randomized either to the follow-up group or to the RAI ablation group (1.1 GBq following rh TSH stimulation). The final estimated study completion date is May 2022. (There is another ongoing prospective trial evaluating the impact of routine RAI ablation in the outcomes of low-risk patients with DTC. The IoN trial [NCT01398085]) is in the phase 2, and Estimated Study Completion Date is March 2031.

Meanwhile, specifically for patients with low-risk DTC for whom the benefits of RAI are not yet clearly established, RAI should be used with caution to minimize any possible harm from treatment, including chronic sialadenitis and secondary malignancies (16,56,57). When ablation is not chosen, the local team must be able to monitor their patients with neck US and serial Tg measurements, as low level Tg values usually remain detectable during follow-up even in the absence of persistent or distant disease (58).

#### Intermediate-risk DTC

Intermediate-risk tumors, the second most common category of DTC (25%-35% of cases), are characterized by tumors with one or more of the following features: presence of minimal extrathyroidal invasion or vascular invasion, aggressive histology, or clinically significant metastatic LNs (clinical N1 or > 5 LNs with size < 3 cm in the largest dimension) (4).

This category includes a very heterogeneous group of tumors with varying degrees of aggressiveness. Variations in recurrence and persistence rates are relatively large in this group (>5%-20%) (Table 1) (4,59,60). Although the risk of persistent disease and recurrence is higher in this group than in the low-risk DTC, the risk of cancer-related death is still low (59).

For intermediate-risk patients, the literature is controversial and there is no consensus on the indication and benefits of adjuvant therapy with ^131^I, as there is insufficient data on the long-term prognosis of these patients (11,61,62). Some retrospective studies have shown that RIT can reduce disease-specific recurrence and mortality in patients of this group (63,64). However, other studies have demonstrated that the benefit of RIT only occurs in a select group of patients in this category.

For example, Buffet and cols. demonstrated the benefit of RIT in reducing the risk of recurrence in cases of aggressive variants of papillary thyroid carcinoma (PTC) (65). Wang and cols. demonstrated that RIT improved DSS only in male patients, patients with age ≥ 45 years, and patients whose tumor size was > 20 mm (66). Interestingly, age has been considered an indicator for response to RIT by some investigators but not by others. Podnos and cols. (67) and Jonklaas and cols. (50) suggested that RIT may benefit older, but not younger, intermediate-risk patients. Meanwhile Ruel and cols. (61) demonstrated that RIT reduced the risk of death in all intermediate-risk patients with PTC.

**Figure 2:**
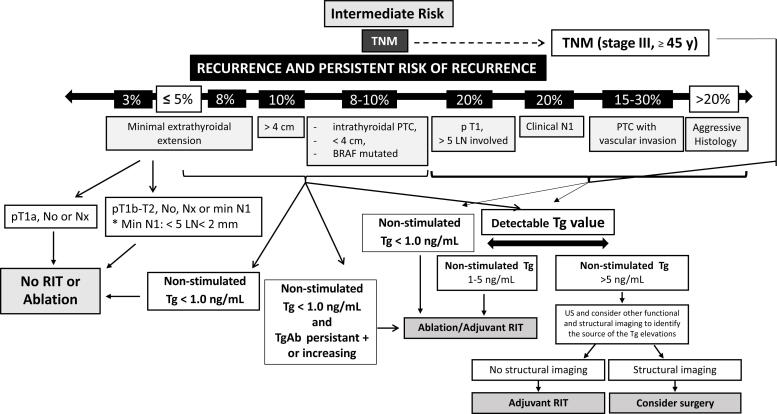
A suggestion for the management of intermediate risk patients.

Both the ATA thyroid cancer guideline task force and the intersocietal working group endorse selective use of RAI for ablation or adjuvant therapy in ATA intermediate risk patients. Individual decision making requires consideration of all the faces of the polyhedron (Figure 1) to reach recommendations supported by the local disease management team for each patient (11,12). The Figure 2 is a suggestion for the management of patients of this category.

#### High-risk DTC

Patients with high-risk DTC account for 5-10% of all patients with DTC. The highest number of disease-specific deaths and recurrence occur in this group (Table 1). Therefore, it is not surprising that these patients require more aggressive treatment and more frequent follow-up than low-risk patients (59). The initial treatment aims to eradicate the disease with total thyroidectomy. Therapeutic LN dissection is recommended for cases with known LN metastases. Prophylactic central neck dissection is advocated by some as it can reduce the risk of disease recurrence (68). RIT is also generally recommended for these cases. The 2015 ATA guideline, the European guideline, and the European Society of Medical Oncology recommend RAI therapy in this group (7,69). In fact, most of the studies analyzing high-risk patients with DTC show a survival benefit and improved recurrence rates after RIT (50,52,67,70,71).

### What is the recommended RAI activity?

Although RIT for DTC has been used successfully for more than 70 years, there is still a lot of controversy regarding the dose of ^131^I to be prescribed in each case. Due to the dose-dependent side effects associated with RAI, ranging from xerostomia and salivary gland dysfunction to secondary malignancy, recent efforts have focused on determining the ideal administered activity that will achieve tumoricidal effects while minimizing the side effect profile (72). The RAI activity can be determined empirically or through dosimetry protocols (73).

#### Empirical RAI activity

Most thyroid cancer disease management teams around the world use a standardized, empirical RAI administered activity which is based on the goals of therapy (adjuvant treatment, remnant ablation, or treatment of known disease) and occasionally modified based on age, renal function, cardiac function, and extent of disease (74). Specifically, for the purpose of ablation, meta-analyses have shown similar success rates when comparing the results of high and low administered activities of ^131^I (74,75).

The ATA 2015 guideline recommends an empirical activity of 30 mCi for remnant ablation. This recommendation is based on two large, randomized studies that compared the efficacy of doses of 30 and 100 mCi for ablation and found that doses of 30 mCi are sufficient, even if using recombinant human thyroid-stimulating hormone (rh TSH) (76,77). However, long-term follow-up studies are needed to demonstrate that the lower ablation administered activity is associated with the very low long term recurrence rates expected in these lower risk patients (73).

For adjuvant therapy, the recommendation of the ATA 2015 guideline is a dosage up to 150 mCi (5.55 GBq) (4). A recent study suggests that intermediate-dose RAI ablation is sufficient as compared to high-dose for adjuvant therapy (78). Sacks and cols., in a subgroup analysis of intermediate- and high-risk patients, demonstrated that the risk of recurrence was not significantly different across a range of RAI administered activities (52).

However, some authors argue in favor of administrating higher administered activities for adjuvant therapy. Jeong and cols. conducted a retrospective study evaluating 204 intermediate-risk DTC patients who underwent post-operative RAI therapy. Patients received 100 and 150 mCi (higher doses) of RAI in one center and 30 mCi (low doses) in the other center. The study showed that low doses of RAI therapy after thyroidectomy appear to be insufficient for intermediate-risk DTC patients. Patients who received low doses demonstrated biochemical or structural incomplete responses to initial RAI therapy and ultimately required more treatment (79).

With respect to the tumoricidal activity in cervical or mediastinal lymph nodes, RAI activity in the range of 150-200 mCi (5.55-7.4 GBq) is suggested. For pulmonary and bone metastases, RAI activity of 100-200 mCi (3.7-7.4 GBq) are usually recommended except for patients > 70 years old in which lower doses are usually given (100-150 mCi (3.7-5.55 GBq) (4). The Society of Nuclear Medicine and Molecular Imaging (SNMMI) suggests a higher activity of 200 mCi for cases with distant metastases (80).

As there is limited long-term data on the impact of various activities for adjuvant and therapeutic RAI activity, it is difficult to determine the optimal dose (81). The administered dose should be based on the multidisciplinary team's management recommendations after consideration of the individual's risks and the benefits.

#### Dosimetric protocols

Although the empiric fixed-dose method is widely used, this method does not evaluate the rate of clearance of RAI from the blood and body or specific individual lesional dosimetry and as a result, the optimal tumoricidal administered activity for an individual patient is not precisely known. Dosimetry based approaches may be particularly useful for pediatric patients, elderly patients, patients with renal insufficiency and patients with RAI avid metastatic lesions. It is also indicated in patients with diffuse lung metastases and in those with unresectable metastatic disease (82).

Whole body and blood dosimetry uses measurements of retained RAI from blood and whole-body measurements done over several days after administration of a tracer dose of ^131^I to determine the maximal tolerable activity (MTA) that can be safely administered to a specific patient. This method was originally reported by Benua and cols. (83) and it limits the absorbed dose to the blood to 200 rad (2 Gy) and 80 mCi to the lung at 48 hours. The objective is to avoid severe damage to the hematopoietic and pulmonary systems. This practice is based on the concept that a higher and safely prescribed activity may have greater therapeutic efficacy and avoid the necessity of repeated treatments with lower doses that can cause non-lethal lesions in cancerous tissues with subsequent cell repair (82).

A meta-analysis showed a correlation between the absorbed dose delivered and therapeutic response, indicating that dosimetry could improve outcomes and survival (84). However, a recent study did not show any difference in the overall survival between patients with distant metastases treated with and without dosimetry. Deandreis and cols. retrospectively analyzed a total of 352 patients with RAI-avid metastatic DTC treated with ^131^I by an empiric fixed activity. A total of 231 patients received 100 mCi (3.7 GBq) at Gustave Roussy and 121 patients were treated with a personalized activity 72-50,2 mCi (2.7-18.6 GBq) based on whole-body dosimetric study at Memorial Sloan Kettering Cancer Center. No benefit in OS was observed in the patients who received personalized activity (85).

Lesional dosimetry is designed to determine the activity of RAI that needs to be administered to achieve a tumoricidal radiation dose to individual metastatic lesions. Maxon and cols. considered the effective dose absorbed by thyroid remnants as 300 Gy and by metastatic disease as 80 Gy. Recent studies have correlated patient outcome with the absorbed dose to the target tissue (86–88). However, to date, the role of dosimetry is still unclear (88,89).

In the absence of definitive proof of superiority of lesional and whole-body RAI clearance-based dosimetry studies, most centers rely on empiric RAI dosing for the vast majority of their patients. Furthermore, dosimetric protocols are complex, laborious, and expensive, which makes it even less likely that these dosimetry approaches would be widely applied at centers around the world.

### How to prepare a patient for RIT

Several known biological phenomena limit the efficacy of RAI therapy. The most challenging factor is the iodine uptake capacity of thyroid cancer tissue, which is poorer than that of normal thyroid cells. To maximize RAI uptake by residual thyroid remnants, persistent disease, or metastases, TSH levels should be increased (above 30 μU/mL) prior to treatment and the whole-body iodine pool should be depleted through a low iodine diet (LID) to decrease the competition between non-RAI and RAI by the NIS (91). A 2 to 3-fold increase in ^131^I uptake by the thyroid is seen following a proper LID (90).

The stringency and duration of iodine restriction are not well-established but most existing studies on the efficacy of the LID limit daily iodine intake to < 50 mcg/day. The duration of the LID is also not standardized and varies according to several major guidelines (4,7,17,69). In areas where the average estimated iodine intake is high, individuals may require a longer duration of iodine restriction (92).

While diet models vary between services, most prohibit foods such as seafood and dairy products. Most also recommend avoidance of canned foods as well as restaurant food (93). An overview of the LID is demonstrated in Table 2.

**Tabla 2:** A Proposed low-iodine diet (LIID) (91)

Restrict iodine diet	
Allowed:	Restricted:
1. Non-iodized salt, snacks and potato chips	1. Iodized salt
2. Fish, seafood, shrimp, oysters and algae	2. Fresh water fish
3. Milk, ice cream, cottage cheese, yogurt, cheese, tofu, soy milk	3. Skimmed milk powder, unsalted butter and margarine
4. Smoked meat, broth, ham, bacon, sausage, sauerkraut	4. Fresh meat
5. Egg yolk, mayonnaise, soy sauce	5. Egg whites, spices, oil, olive oil, vinegar
6. Canned fruit, fruit in syrup, salted nuts	6. Fresh fruits and juices, fruit with salt, nuts and peanuts
7. Watercress, celery, Brussels sprouts, cabbage, olives, pickles, mushrooms	7. Lettuce, beets, broccoli, onion, carrot, cabbage flower, peas, spinach turnip, cucumber, tomato, pod
8. Industrialized breads, pizza	8. French bread, cream cracker, spaghetti, rice, oats, barley Flour, beans, corn, wheat
9. Chocolate, milk, red candies	9. Sugar, honey, jam, candy
10. Tea	10. Juice and soda

A spot urinary iodine concentration (UIC) is a convenient marker of iodine depletion and despite the absence of guidelines on the routine evaluation of urinary iodine levels, a spot UIC < 100 mcg/L rules out significant iodine contamination and can be used as verification of adequate LID preparation (93).

### When is RAI no longer likely to be effective therapy?

Most patients with DTC have a good prognosis with high cure rates after initial treatment. However, in some cases, the disease may persist or recur, and additional treatments with ^131^I may be necessary (94). The ATA 2015 guidelines suggest that ^131^I-avid metastatic lesions can be treated with ^131^I and that RIT can be repeated when an objective benefit is demonstrated (4,95). Many centers continue RIT as long as there are ^131^I-avid lesions on Rx-WBS and/or elevated Tg and/or until a maximum dose of 600 mCi is reached (96).

As new treatment options emerge for radioiodine refractory (RAIR)- DTC patients, it is important to recognize when RIT is no longer beneficial.

The phenomenon of iodine refractoriness was first reported in 1952 by Paterson and cols. (97). Although the concept of RAIR-DTC has been around for years, there is still no consensus regarding its definition. This is due to the fact that there is no single criterion for RAIR-DTC, but rather, conditions that increase the likelihood of this diagnosis (12). Continuous improvement in molecular imaging and molecular pathology will likely permit a more accurate definition, but in the moment, variations in the definition of RAIR-DTC persist in practice and in clinical trials design (4,12,98).

- No RAI uptake is present on a diagnostic RAI scan
- **No RAI update is present on a RAI scan performed after RAI therapy**
- RAI uptake is present in some but not other tumor foci
- Metastatic disease progresses despite RAI uptake
- Metastatic disease progresses despite a cumulative ^131^I activity of > 600 mCi (> 22.2 GBq) (96)

However, a cumulative ^131^I activity of > 600 mCi is not an established definition for iodine refractoriness; many centers continue RIT as long as they are ^131^I-avid lesions on Rx-WBS and/or elevated Tg and/or until a maximum dose of 600 mCi is reached. A maximum dose of 600 mCi emerged from a study of Durante and cols. A total of 444 patients with distant metastases from papillary and follicular thyroid carcinoma were evaluated. All patients were treated with 3.7 GBq (100 mCi) after withdrawal of thyroid hormone treatment every 3-9 months during the first 2 years and then annually until the disappearance of any metastatic uptake. Most negative studies were obtained with cumulative activities lower than or equal to 22 GBq (600 mCi) (96). Additionally, previous studies have shown that the risk of secondary malignancy, including leukemia, becomes significant with cumulative activities greater than 22 GBq.

While these common clinical scenarios have traditionally been used to identify patients likely to be RAI refractory, newer studies demonstrating that a few weeks of targeted therapy administration can restore RAI uptake in metastatic foci previously classified as RAI refractory are expected to make us re-think our criteria to classify as patient as RAI refractory. Specifically, selumetinib (99) (a selective MEK inhibitor) and dabrafenib (a selective BRAF inhibitor) (100) have been shown to increase RAI uptake by RAIR tumors.

In conclusion, decision making regarding the role of RAI therapy in differentiated thyroid cancer has gotten exponentially more complicated over the last decade. The dramatic rise in the diagnosis of many low-risk thyroid cancers has made us re-evaluate our one size fits all approach to thyroid cancer therapy. As noted above, individual decision making requires the patient and the clinician to consider a host of factors represented by the multiple faces of the polyhedron (Figure 1) to arrive at the “best” management recommendation for a particular patient within their specific clinical context and respecting the patients’ expectations, beliefs, priorities, and preferences.
